# CRISPR-Cas12a test strip (CRISPR/CAST) package: In-situ detection of Brucella from infected livestock

**DOI:** 10.1186/s12917-023-03767-1

**Published:** 2023-10-13

**Authors:** Sheng Dang, Humujile Sui, Shuai Zhang, Dongxing Wu, Zeliang Chen, Jingbo Zhai, Meirong Bai

**Affiliations:** 1 Innovative Institute of Zoonoses, Inner Mongolia Minzu University, Tongliao, 028000 China; 2 Key Laboratory of Zoonose Prevention and Control at Universities of Inner Mongolia Autonomous Region, Tongliao, 028000 China; 3 Brucellosis Prevention and Treatment Engineering Research Center of Inner Mongolia Autonomous Region, Tongliao, 028000 China; 4 Mongolian Medical College, Inner Mongolia Minzu University, Tongliao, 028000 China; 5 Key Laboratory of Mongolian Medicine Research and Development Engineering, Ministry of Education, Tongliao, 028000 China

**Keywords:** *Brucella* infection, CRISPR-Cas12a, RPA, Test strip, CRISPR/CAST package

## Abstract

**Background:**

Brucellosis is a common zoonotic disease caused by *Brucella*, which causes enormous economic losses and public burden to epidemic areas. Early and precise diagnosis and timely culling of infected animals are crucial to prevent the infection and spread of *Brucella*. In recent years, RNA-guided CRISPR/Cas12a(Clustered Regularly Interspaced Short Palindromic Repeats and its associated protein 12a) nucleases have shown great promise in nucleic acid detection. This research aims to develop a CRISPR/CAST (CRISPR/Cas12a Test strip) package that can rapidly detect *Brucella* nucleic acid during on-site screening, especially on remote family pastures. The CRISPR/Cas12a system combined with recombinase polymerase amplification (RPA), and lateral flow read-out.

**Results:**

We selected the conserved gene *bp26*, which commonly used in *Brucella* infection detection and compared on Genbank with other *Brucella* species. The genomes of *Brucella abortus* 2308, *Brucella suis* S2, *Brucella melitansis* 16 M, and *Brucella suis* 1330, et al. were aligned, and the sequences were found to be consistent. Therefore, the experiments were only performed on *B. melitensis*. With the CRISPR/CAST package, the assay of *Brucella* nucleic acid can be completed within 30 min under isothermal temperature conditions, with a sensitivity of 10 copies/μl. Additionally, no antigen cross-reaction was observed against *Yersinia enterocolitica* O:9, *Escherichia coli* O157, *Salmonella enterica serovar* Urbana O:30, and *Francisella tularensis*. The serum samples of 398 sheep and 100 cattle were tested by the CRISPR/CAST package, of which 31 sheep and 8 cattle were *Brucella* DNA positive. The detection rate was consistent with the qPCR results and higher than that of the Rose Bengal Test (RBT, 19 sheep and 5 cattle were serum positive).

**Conclusions:**

The CRISPR/CAST package can accurately detect *Brucella* DNA in infected livestock within 30 min and exhibits several advantages, including simplicity, speed, high sensitivity, and strong specificity with no window period. In addition, no expensive equipment, standard laboratory, or professional operators are needed for the package. It is an effective tool for screening in the field and obtaining early, rapid diagnoses of *Brucella* infection. The package is an efficient tool for preventing and controlling epidemics.

**Supplementary Information:**

The online version contains supplementary material available at 10.1186/s12917-023-03767-1

## Background

The global burden of *Brucella* infection in livestock is substantial, and conservative estimates are that 300 million of the 1.4 billion cattle population worldwide are infected with the organism [1]. *Brucella* threatens the breeding industry and human health and seriously affects the import and export trade of livestock and meat, causing very large economic losses and social burdens. Even in developed countries, *Brucella* infection cannot be completely eradicated because infected wildlife can persistently become infected and spread the disease to domestic animals [2, 3]. Currently, serological detection is the primary method to diagnose brucellosis, and this method depends on the detection of infected livestock-specific antibodies, indirectly revealing the presence of this pathogen. As the history of *Brucella* exposure, animal immune system, immune response, and resulting patterns of antibody generation and dynamics are variable among individuals, no direct evidence can prove the presence of the pathogen [2]. RBT is a card agglutination test that is simple to operate, rapid and exhibits high sensitivity. The method is suitable for field assays and is usually used as a screening test for brucellosis [4]. Nonetheless, *Brucella* contains antigens that cross-react with *Yersinia enterocolitica* O:9*, Escherichia coli* O157*, Salmonella enterica serovar* Urbana O:30*,* and *Francisella tularensis*, easily leading to false-positives. Therefore, the serological test of brucellosis does not provide objective, direct diagnostic evidence [5].

Moreover, RBT cannot detect *Brucella* in infected livestock during the window period. PCR is a practical method that can amplify nucleic acids for detecting sample-infected microorganisms [6, 7], and PCR exhibits high sensitivity [8]. However, PCR-based methods have many limitations, such as expensive instruments, matching standard reagents, reliance on high-standard laboratories and professional technicians, and diagnostic standards that are not yet unified. Developing countries and rural areas with high brucellosis morbidity cannot easily implement PCR, as the countries lack laboratory equipment and professional technicians. Furthermore, PCR is time-consuming and not suitable for rapid on-site screening [9]. Collectively, faster and more effective methods for assaying nucleic acids in the field are urgently needed.

In recent years, RNA-based guide CRISPR/Cas nucleases have shown great promise in detecting nucleic acids with high sensitivity and rapidity. This system has been transformed into an efficient gene-editing tool that is widely applied for gene editing in eukaryotes [10]. Recently, scientists have found that some class II Cas proteins, such as Cas12a (Cpf1), exhibit accessory activity in cleaving single-stranded DNA (ssDNA), and the CRISPR‒Cas system has been applied to the nucleic acid assay field [11]. Zhang et al. discovered the CRISPR-Cpf1 system [12], which is mediated by a single Cas protein with cleavage activity similar to the CRISPR‒Cas9 system in 2015. Compared to Cas9, Cpf1 is a single RNA-guided endonuclease that lacks tracrRNA, recognizing T-rich PAM (protospacer-adjacent motif), and Cpf1 introduces a staggered DNA double-stranded break with a 4- or 5-nt 5' overhang. With guidance by crRNA, Cas12a binds to the target sequence and cleaves the target double-stranded DNA; however, Cas12a-induced cleavage of single-stranded DNA in the system is arbitrary. According to this discovery, Doudna et al. developed a diagnostic system named DETECTR (DNA Endonuclease Targeted CRISPR Trans Reporter) in 2018 [11]. This system can quickly and instantly detect trace amounts of DNA in samples. DETECTR is a nucleic acid detection system that combines isothermal amplification, CRISPR‒Cas12a, crRNA, and fluorescent reporter groups; with this system, human papillomavirus (HPV) was successfully detected. Visualization is essential for the wide application of nucleic acid assays. In the same year, Zhang et al. developed "SHERLOCKv2" (Specific High-sensitivity Enzymatic Reporter un-LOCKing Version 2) [13], which uses lateral flow strips that produce color changes, which are observed by the naked eye. Several CRISPR‒Cas based methods have been developed to detect and diagnose infectious and noninfectious diseases (such as cancer) [14–16]. Compared to PCR, DETECTR and SHERLOCKv2 provide another level of ultrasensitive detection method [17]. Recombinase polymerase amplification (RPA) is a new nucleic acid isothermal amplification technology that was developed in 2006 [18]; this technology can exponentially amplify template DNA in a short time at an isothermal temperature. Therefore, RPA can eliminate the dependence on the PCR instrument. Similar to PCR, RPA also showed nonspecific amplification [19]. Thus, by combining the RPA with the CRISPR‒Cas12a system, the single-stranded DNA containing a reporter group is added to the system and target fragment amplification is achieved through RPA. Cas12a recognizes and binds to the amplification product and then cleaves the single-stranded probe within the system to release the fluorescent reporter group. Finally, target DNA can be detected by capturing the fluorescent signal. The nucleic acid detection test strip adopts a chromatographic double-antibody sandwich method to detect the probes cleaved by Cas12a. When designing probes, one end of ssDNA is labeled with biotin and the other with 6-carboxyfluorescein (6-FAM) or fluorescein isothiocyanate (FITC). The target fragment of *Brucella* DNA is amplified through isothermal amplification (e.g., RPA, LAMP, RAA), and then the amplification products and the labeled ssDNA with the fluorescent reporter group are cleaved by Cas12a simultaneously. As a result, the test strip can detect the fluorescent signal to identify *Brucella* infection. The method exhibits high sensitivity and strong specificity and thus provides a new method for earlier and more rapid on-site diagnoses of *Brucella*.

**Fig. 1:**
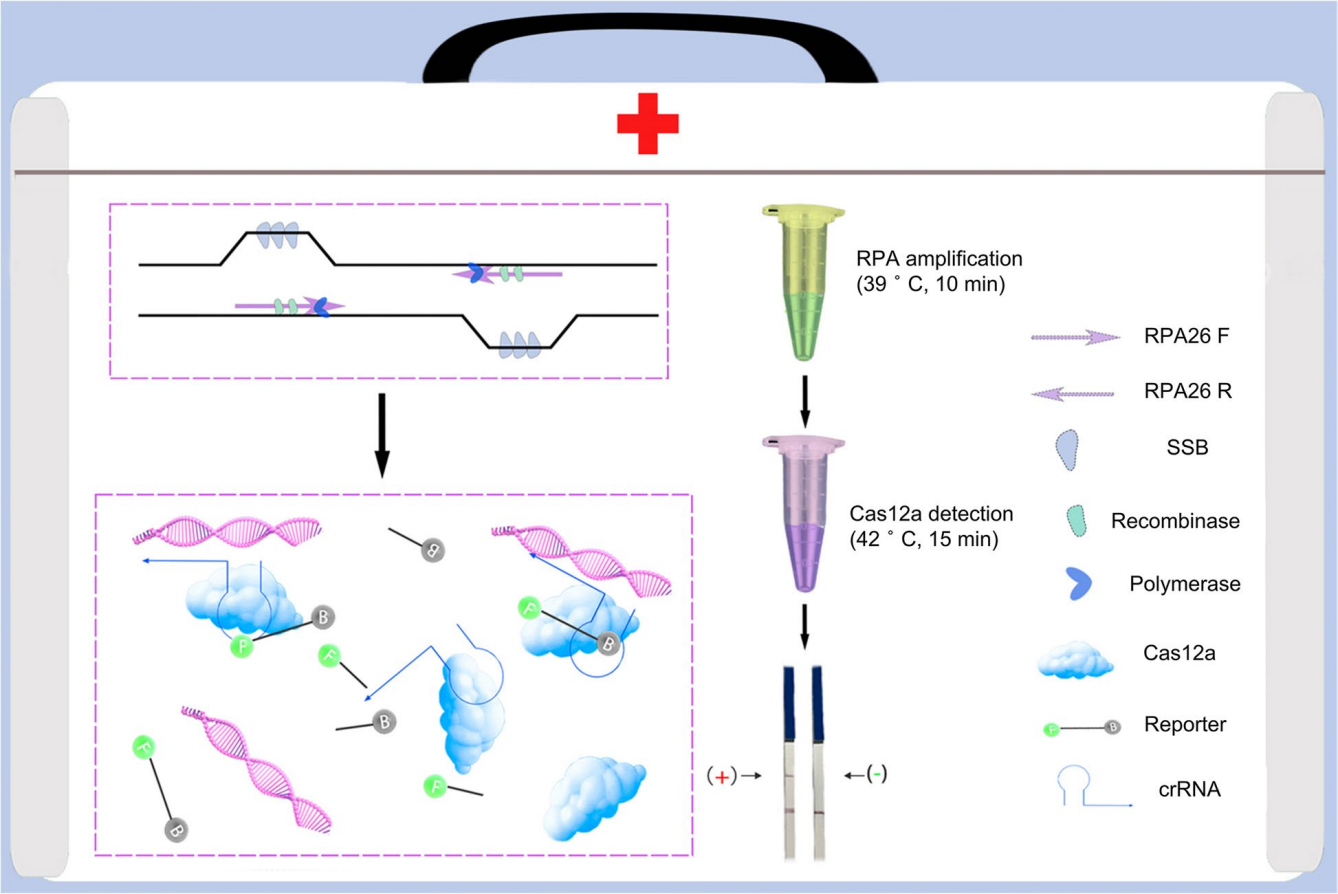
The CRISPR/CAST package detection principle. Figure 1 shows the detection principle for the CRISPR/CAST package, and the detection process is divided into three steps. In the first step, the RPA isothermal amplification technology is used to amplify the *Brucella* target DNA. In the second step, 3 μl RPA amplification products are added to the reaction solution containing Cas12a, crRNA and the ssDNA with fluorescent report group at 42 °C for 10 min. The third step is to dilute the second reaction solution with pure water to 50 μl and then insert the test strip into the solution. After the test strip is thoroughly infiltrated, the result can be observed within 10 min with the naked eye. If the detection line color is present, the result is positive. The result is negative if the detection line shows no color and the quality control line is present. The result is invalid if the quality control and detection line color are present

This study refers to the principle of the DETECTR and SHERLOCKv2 systems, which combines the CRISPR‒Cas12a system and RPA, by observing lateral flow strip colors for assay the result. This method is more convenient for on-site detection, and the *Brucella* rapid CRISPR/CAST package was thus built. The method is simple and exhibits high sensitivity and strong specificity; in addition, the *Brucella* nucleic acid assay only takes approximately 30 min, rapidly detecting *Brucella* nucleic acid during on-site screening, which is especially useful on remote family pastures (Fig. 1).

## Results

### crRNA screening

**Fig. 2:**
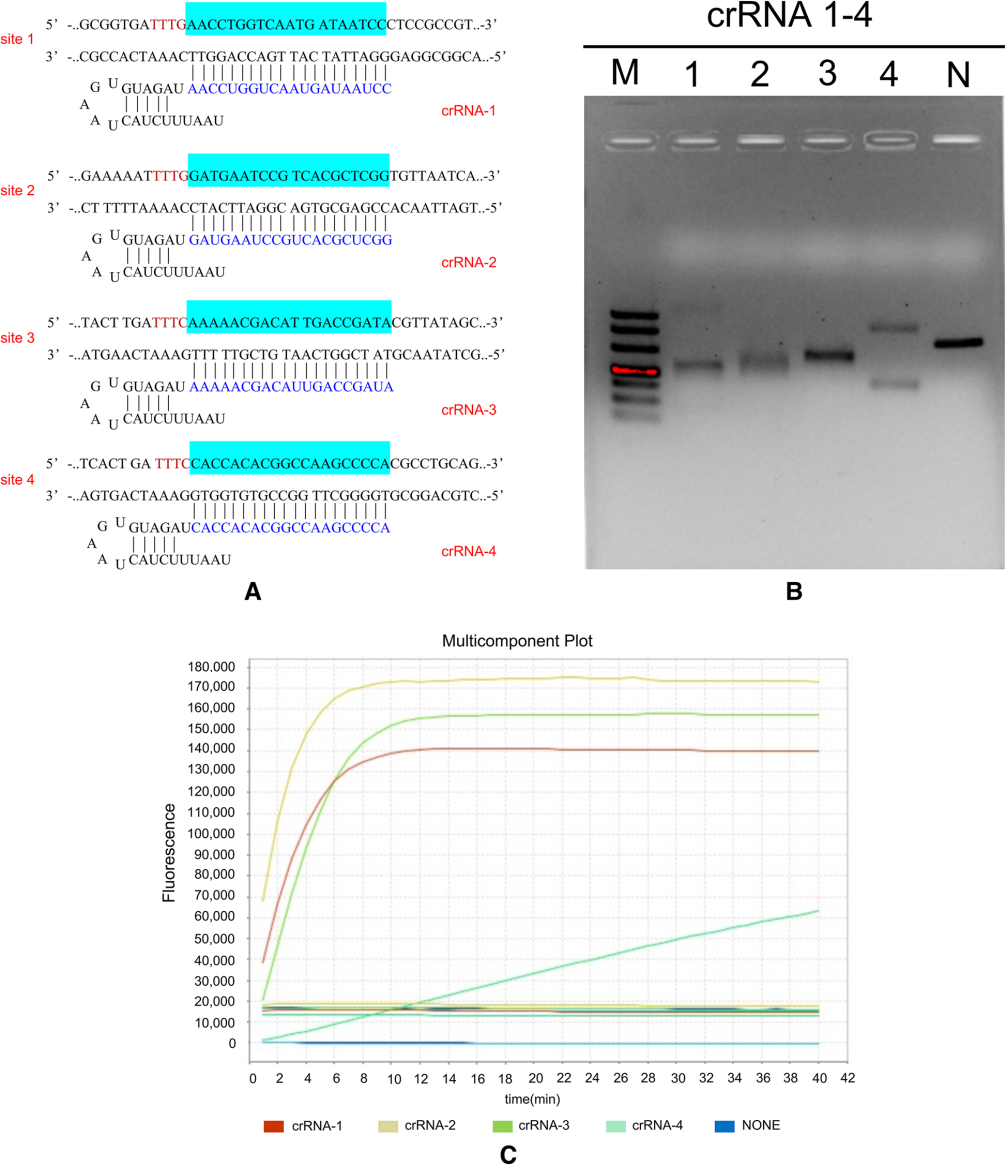
crRNA screening. **A** The bp26-crRNA-1–4 structure and the corresponding target sequences. The target sites are highlighted in blue, and PAM sequences are marked in red. **B** To determine the products of Cas12a cleavage, 20 μl of each reaction was electrophoresed (2% agarose), M: DL 500 DNA marker (Takara, Code No. 3590Q), 1–4: crRNA-1–4, (RPA amplification products were 340 bp, 261/79 for crRNA-1, 303/37 for crRNA-2, 310/30 for crRNA-3, 174/166 for crRNA-4), N: negative control. **C** Four groups of crRNAs were screened by the quantitative fluorescence method. The reaction was performed at 42 °C for 40 min, and the signal was collected every 1 min. The reaction entered the plateau phase at 8–10 min. As shown in the figure, the endpoint fluorescence value of crRNA-2 was slightly higher than that of crRNA-1 and crRNA-3 and significantly higher than that of crRNA-4

Four groups of crRNAs (bp26-crRNA-1–4) (Table 1) were compared by the RPA-Cas12a method, and four groups of crRNA binding sites are indicated below (Fig. 2A). The Cas12a cleavage products were examined by electrophoresis on a 2% agarose gel to exhibit the cleavage efficiency (Fig. 2B). Moreover, as shown in Fig. 2C, the endpoint fluorescence value was obtained with a real-time PCR instrument. The most efficient crRNA was selected by the above two approaches. The gel electrophoresis of bp26-crRNA-1, 2 and 3 Cas12a digestion products showed complete cleavage compared to that of bp26-crRNA-4 (Fig. 2B). The real-time fluorescence quantitative PCR results indicated that the endpoint of bp26-crRNA-2 produced the highest value (Fig. 2C). Therefore, all subsequent experiments were performed by adopting bp26-crRNA-2 (uaauuucuacuaaguguagauGAUGAAUCCGUCACGCUCGG).

**Table 1:** The crRNAs sequence

Category	crRNAs name	crRNAs sequence (5′ → 3′)
*bp26*	bp26-crRNA-1	uaauuucuacuaaguguagauAACCUGGUCAAUGAUAAUCC
	bp26-crRNA-2	uaauuucuacuaaguguagauGAUGAAUCCGUCACGCUCGG
	bp26-crRNA-3	uaauuucuacuaaguguagauAAAAACGACAUUGACCGAUA
	bp26-crRNA-4	uaauuucuacuaaguguagauCACCACACGGCCAAGCCCCA

### CRISPR/CAST package sensitivity test

**Fig. 3:**
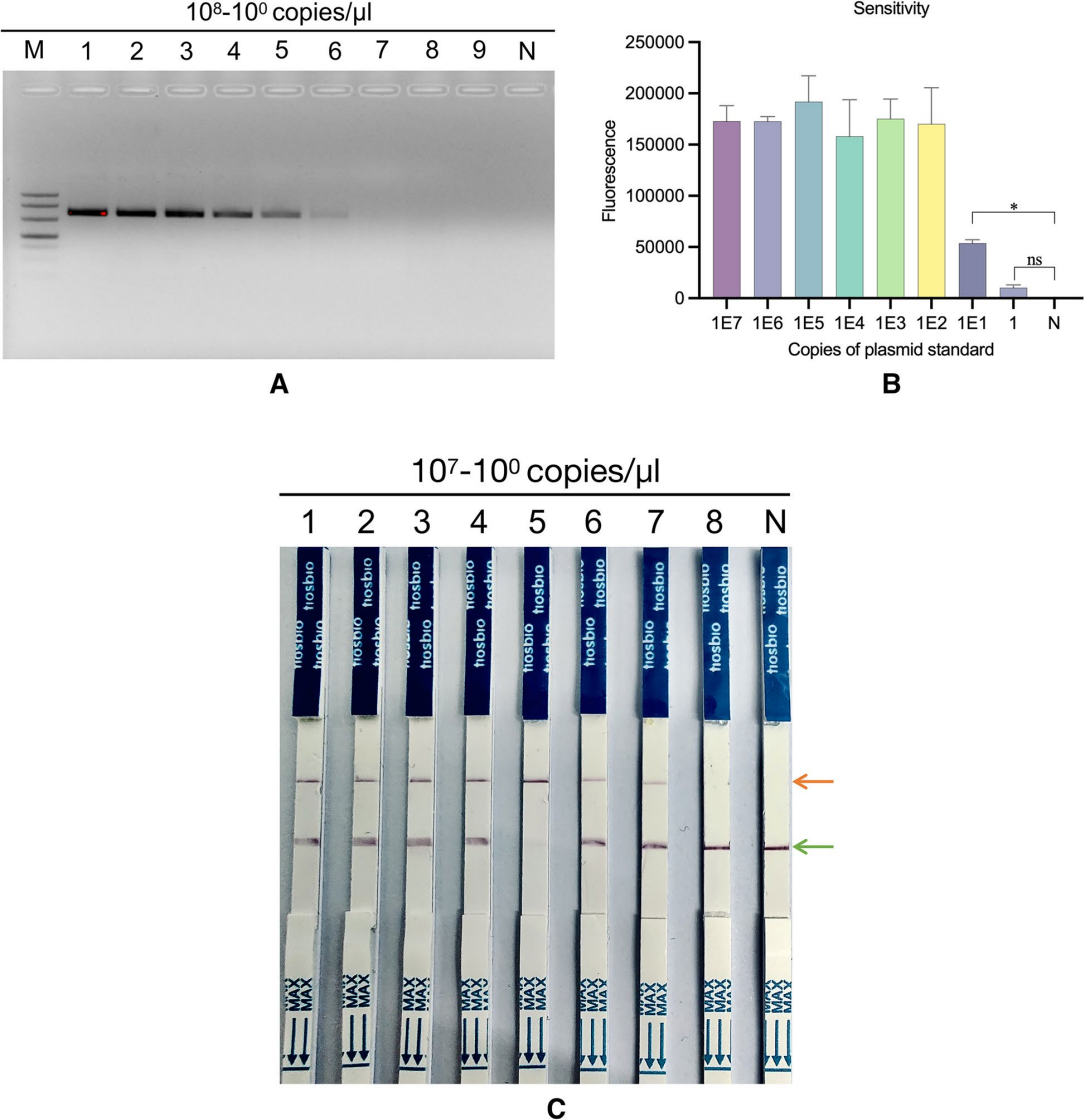
Sensitivity of the three methods. **A** Sensitivity of the standard RPA for nucleic acid detection. After amplification at 39 °C for 15 min, 5 μl of each reaction was electrophoresed (2% agarose), M: 500 bp Marker (Takara, Code No. 3590Q), 1–8 corresponds to 10^8^–10^0^ copies/μl plasmid standard, N: a negative control. **B** Sensitivity of the DETECTR method. A volume of 3 μl of standard RPA product was added to the Cas12a cleavage system, and maximum fluorescent signals were recorded (*n* = 3, error bars show the mean ± SEM). **C** Sensitivity of the CRISPR/CAST package, 1–8 corresponding to plasmid copy numbers of 10^7^–10^0^ copies/μl, N: negative control, the green arrow is the quality control line, and the orange arrow is the detection line

The RPA sensitivity test prepared a series of diluted positive quality controls with 10^8^ − 10^0^ copies/μl as the template and RNase-free water as the negative control. The CRISPR/CAST package sensitivity test prepared a series of diluted positive quality controls with 10^7^ − 10^0^ copies/μl as the template and RNase-free water as the negative control. The results are shown in Fig. 3. The lower detection limit for RPA was 1000 copies/μl (Fig. 3A), and the CRISPR/CAST package sensitivity test was 10 copies/μl (Fig. 3B, C).

### CRISPR/CAST package specificity test

**Fig. 4:**
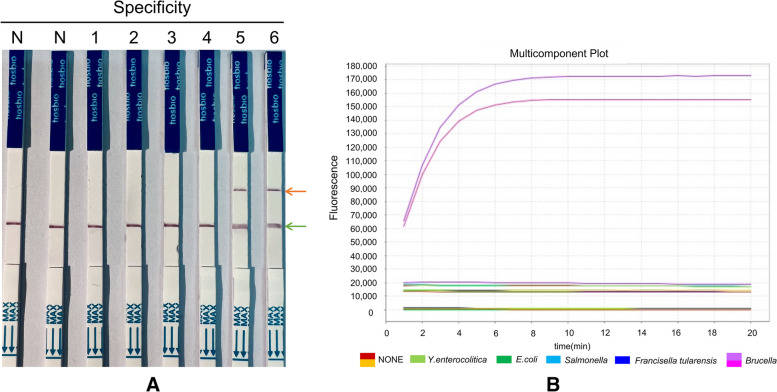
The specificity test. **A** Specific test strip test. N: negative control, 1–4 are *Yersinia enterocolitica* O:9*, Escherichia coli* O157*, Salmonella enterica serovar* Urbana O:30*,* and *Francisella tularensis*, 5,6 *Brucella*; the green arrow is the quality control line, and the orange arrow is the detection line. **B** Specificity was detected by the fluorescence quantification method

Primers and crRNA sequences were compared with *Brucella* and its cross-strains (*Yersinia enterocolitica* O:9, *Escherichia coli* O157, *Salmonella enterica serovar* Urbana O:30, and *Francisella tularensis*) by BLAST function. The RPA primers and four groups of crRNA alignments were single copies of the *Brucella* gene, and cross strains exhibited no matching fragments. The *Brucella* DNA sample test was positive, negative controls (no RNA enzyme water) and *Yersinia enterocolitica* O:9*, Escherichia coli* O157*, Salmonella enterica serovar* Urbana O:30*,* and *Francisella tularensis* samples were all negative (Fig. 4). The above results showed that the CRISPR/CAST package could specifically assay target genes in the *Brucella* DNA samples without cross-reacting with other bacterial nucleic acids, indicating that the assay showed high specificity and that the samples of *Yersinia enterocolitica* O:9*, Escherichia coli* O157*, Salmonella enterica serovar* Urbana O:30*,* and *Francisella tularensis* showed no false-positive results.

### Effect evaluation of the serum samples test

Blood samples from 398 sheep and 100 cattle from the Tongliao Zarut Banner area were tested by RBT. DNA was extracted from serum samples and the CRISPR/CAST package and qPCR were performed to assay *Brucella* DNA. The results are shown in Table 2, in which 31 sheep and 8 cattle were *Brucella* DNA positive. The detection rate was consistent with the qPCR results and higher than the RBT results (19 sheep and 5 cattle serum samples were positive).

**Table 2:** Serum samples test

Sample type	Serum of 398 sheep			Serum of 100 cattle		
test method	CRISPR/CAST package	qPCR	RBT	CRISPR/CAST package	qPCR	RBT
Positive	31	31	19	8	8	5
Negative	367	367	379	92	92	95

## Discussion

Infections caused by *Brucella* have emerged as a considerable threat worldwide, particularly in vast amounts of livestock. It is essential to eradicate and control *Brucella* infections in livestock from the source. In addition, earlier detection and timely culling are equally important. Consequently, the screening used for livestock must be accurate, sensitive, specific, simple, and fast. At present, the methods of diagnosing brucellosis mainly include agglutination tests, real-time PCR, ELISA, semiquantitative PCR, colloidal gold test strips, and polarized light technology. No single diagnostic method can meet the required sensitivity and specificity criteria. Some methods exhibit low specificity and sensitivity, such as agglutination and colloidal gold test strips. Some require special equipment, complex procedures, and professional personnel. Therefore, these methods, such as real-time PCR, can only be carried out in professional laboratories and are unsuitable for on-site testing by herders. RBT is simple, rapid, and highly sensitive and is the primary method currently used for screening brucellosis in livestock groups; in addition, RBT is the designated test for brucellosis in cattle, sheep, and pigs in international trade [20]. However, the results are subjective, and there are cross-antigens with other bacteria, such as *Brucell*a, *Yersinia enterocolitica* O:9, *Escherichia coli* O157, *Salmonella enterica serovar* Urbana O:30, and *Francisella tularensis*, and cross-agglutination reactions with brucellosis-specific antibodies; thus, the method is prone to false-positive results. Therefore, RBT cannot be used as objective and direct diagnostic tool for brucellosis and cannot assay *Brucella* infection during the window period. That is, the antibodies are not produced at the initial infection stage. RPA is carried out under isothermal conditions, realizing nucleic acid detection independent of professional instruments and personnel. Although RPA is simple to operate and exhibits high amplification efficiency, nonspecific amplification is also inevitable.

As reported, a single RPA cannot assay low levels of targets [13]. These studies have confirmed Cas12a accessory splicing ssDNA activity for nucleic acid detection. The ternary complex consists of *Brucella* DNA, Cas12a, and crRNA. Cas12a possesses a RuvC domain that can exert activity to arbitrarily cleave ssDNA labeled with a fluorescent signal. For real-time PCR and RPA detection, the probe corresponds to the template individually. Theoretically, under identical circumstances, we can hypothesize that the fluorescent signals produced by the CRISPR‒Cas12a reaction are higher than those of real-time PCR and RPA detection. However, how much higher these signals are remains ambiguous, so we need to conduct additional experiments. By detecting *Brucella* DNA with the fluorescence signal, we can determine whether livestock have been infected. The proposed method exhibits high sensitivity and strong specificity. The test strip is portable and convenient for performing field assays. When designing the probe, one end of ssDNA was labeled with biotin, and the other end was labeled with 6-FAM. Through lateral flow chromatography detection, the nucleic acids present during *Brucella* infections can be identified without relying on large-scale instruments (Fig. 1). This study developed a new, rapid, sensitive, and specific nucleic acid detection package (CRISPR/CAST package) that can be used in grassroots veterinary stations and farms. The method is vitally significant for the earlier diagnosis of *Brucella* infection, comprehensive prevention and control, and elimination of the threat of infected animals to environmental biosecurity.

The CRISPR/CAST package combines RPA, CRISPR‒Cas12a, and nucleic acid detection test strips. The assay was completed within 30 min under isothermal conditions, with a sensitivity of 10 copies/μl (Fig. 3B, C). In addition, the CRISPR/CAST package can detect *Brucella* without involving antigenic cross-reactions to *Yersinia enterocolitica* O:9, *Escherichia coli* O157, *Salmonella enterica serovar* Urbana O:30, and *Francisella tularensis* (Fig. 4). The high specificity is due to the specific primers designed in the RPA reaction, which is followed by the specific binding of crRNA to the target sequence. Through this dual-specific base-complementary binding, detection is achieved even if nonspecific amplification occurs in the RPA reaction. The second-step crRNA cannot complementarily pair with the target sequence; as a result, the cleavage reaction of Cas12a does not occur and no fluorescent signal can be collected and illuminated. Cas9 must form a complex with two small RNAs (sgRNA and tracrRNA), both of which are necessary for cleavage activity; however, Cas12a only needs one crRNA to form a complex [21], which increases the efficiency, is flexible in binding to the target sequence and decreases the off-target probability. Logistically, Cas12a presents a more minimalistic system than that of Cas9 [22].

The CRISPR/CAST package exhibits unique advantages in nucleic acid detection. On the one hand, the assay, which is completed in a shorter time (approximately 30 min), is rapid. On the other hand, the package is portable; that is, no large instrument, professional laboratory, or professional and technical personnel are needed; in addition, the package utilizes the strong specificity of the *Brucella* nucleic acid assay and does not involve cross-reactions to other organisms. The specific primers are used during isothermal amplification, and the complex formed by crRNA and Cas12a proteins can be accurately located in the target sequence [23]. This dual localization ensures the high specificity of the CRISPR/CAST package (Fig. 4). The lower detection limit for the CRISPR/CAST package sensitivity experiment was 10 copies/μl (Fig. 3B, C), while that of RPA was 1000 copies/μl (Fig. 3A). Due to the strip, the results are observable by the naked-eye (Figs. 3C, 4A).

Thus, the CRISPR/CAST package is superior to conventional serological methods and PCR for detecting *Brucella* infection. Field serum samples of 398 sheep and 100 cattle were tested by the CRISPR/CAST package, of which 31 sheep and 8 cattle were *Brucella* DNA positive. The detection rate was consistent with the qPCR and higher than that of the RBT (19 sheep, 5 cattle were serum positive). Due to the CRISPR/CAST package, which significantly promotes livestock health, nucleic acid detection technology can be successfully utilized by pastoral households. With the package, infected animals can be screened earlier, culling can be performed in a timely manner and the infected environment can be cleaned.

Additionally, the method can control the incidence effectively, reduce the spread of *Brucella* infection and suppress large-scale infection, improving the quality of meat and dairy products. Furthermore, it ensures the safety of individual farmers' transactions, and it can protect the livestock herd's safety and reduce the probability of abortion in pregnant livestock. Thus, farmers' losses are reduced. Improving the quality of meat and dairy products can also promote the import and export trade and protect human health. The CRISPR/CAST package, which exhibits high sensitivity, strong specificity, and portability, helps farmers and herders who live in remote areas complete the test at their convenience. The CRISPR/CAST package provides a tool for screening in the field, detects *Brucella* infection in an early and rapid manner, and inspires new ideas for establishing rapid nucleic acid assays for other pathogens. We believe that this phenomenal technology will prevent livestock from incurring *Brucella* infection and safeguard the herders' benefits and health indefinitely. The CRISPR‒Cas system is leading to a new technological revolution. This new diagnostic tool will rewrite future diagnostic technologies, especially in developing countries with relatively poor sanitation and a high incidence of animal diseases.

## Conclusions

The CRISPR/CAST package can detect *Brucella* DNA in infected livestock within 30 min. It is simple and rapid, exhibits high sensitivity and strong specificity, has no window period, and does not require expensive equipment, standard laboratories, or professional personnel. The package is an efficient tool for controlling and preventing epidemics.

### Materials

The *Brucella* strain is *B. melitensis* from Kailu County of Tongliao City, Inner Mongolia Autonomous Region, China (GenBank No. CIT21: CP025819, CP025820). The positive quality control plasmid from T-Vector pMD19 inserted target sequence fragments was synthesized in our laboratory and extracted with a TIANprep Mini Plasmid Kit purchased from Tiangen (Beijing, China). A *Brucella* infection pretreatment kit was used [24]. The TwistAmp Basic kit was purchased from TwistDx Ltd. (Hertfordshire, AL, U.K.). The Cas12a protein and lateral flow strip were purchased from Bio-Lifesci (Guangzhou, China). RNase inhibitor was purchased from TaKaRa Bio Inc. (Dalian, China). *Yersinia enterocolitica* O:9*, Escherichia coli* O157*, Salmonella enterica serovar* Urbana O:30*,* and *Francisella tularensis* plasmids were stored in our laboratory.

## Methods

### RPA primer design

In this study, the highly conserved sequence *bp26* (Genbank No. OQ378327) gene was selected as the target sequence from the National Center for Biotechnology Information (NCBI), and then Premier 5.0 software was used to design RPA forward and reverse primers [25] based on primer design principles (Table 3). BLAST (The Basic Local Alignment Search Tool) was used to compare the primer sequences with the genomes of the cross strains *Yersinia enterocolitica* O:9*, Escherichia coli* O157*, Salmonella enterica serovar* Urbana O:30*,* and *Francisella tularensis* to determine the primer specificity. The primers were synthesized by Sangon Biotech (Shanghai, China).

**Table 3:** RPA primers sequence

Gene	Primers name	Primers sequence (5′ → 3′)
*bp26*	RPA26 F	CAATGTTGGAAAAATTTTGGATGAATCCGT
	RPA26 R	TTACTTGATTTCAAAAACGACATTGACCGATA

### crRNA and probe design

According to the LbCas12a protein corresponding to the crRNA stem‒loop structure and the target gene sequence, referring to the crRNA design requirements [26], four groups of specific crRNAs (Table 1) were designed on the *bp26* gene sequence of *Brucella.* We referred to Chen et al. for the reporter probe design [11]. The fluorescent probe (yg-probe) sequence is 6-FAM-TTATT-BHQ. The test strip probe refers to the principal design of nucleic acid test strip detection, and the sequencing probe (szt-probe) is 6-FAM-TTATT-Biotin. crRNAs and probes were synthesized by Sangon Biotech (Shanghai, China).

### CRISPR/CAST package reaction

*Standard RPA reaction assay* (50 μl): 2.4 μl of RPA forward-and-reverse-direction primers F and R (10 μM), 29.5 μl of primer-free rehydration buffer, 2.5 μl of MgOAc (280 mM), 1 μl of target DNA, RNase-free water was replenished to 50 μl. The mixture was incubated in a conventional water bath at 39 °C for 15 min. *Cas12a cleavage assay* (25 μl): 3 μl of 10 × LbCas12a buffer, 1 μl of LbCas12a (500 pmol), 1 μl of crRNA (10 μM), 0.5 μl of RNase inhibitor (5 U/μl), 0.5 μl of szt-Probe or yg-Probe (10 μM), 3 μl of the RPA amplified product, refilling to 25 μl with RNase-free water. The prepared reaction tube was centrifuged at 12,000 r/min for several seconds. The mixture was incubated in a conventional water bath at 42 °C for 10 min. *Test strip detection*: the samples were transferred to a safe area after the Cas12a cleavage reaction and diluted to 50 μl with RNase-free water. Test strip was inserted below the liquid level. After the test strip had infiltrated completely, the test strip was removed, and the result was observed within 10 min with the naked eye.

### Screening of the crRNA

The constructed *Brucella* *bp26* plasmid was used as the template, the CRISPR/CAST package reaction was applied, and four groups of crRNAs (bp26-crRNA-1–4) were screened. The Cas12a cleavage products were be checked by electrophoresis on a 2% agarose gel to observe the cleavage efficiency. Furthermore, the endpoint fluorescence value was observed with a real-time PCR instrument, and the most efficient crRNA was selected by the above two approaches.

### CRISPR/CAST package sensitivity test

The target fragment was amplified by RPA isothermal amplification technology and then recovered by a TIANgel Midi Purification Kit. The target fragment was connected to the pMD®19-T vector and transformed into DH5α Escherichia coli competent cells. The solid LB agar plate with ampicillin resistance was streaked and incubated overnight at 37 °C, after which single colonies were picked and expanded, and finally, the positive plasmid was extracted. The *bp26*-positive plasmid concentration was quantified by a spectrophotometer, SimpliNano (GE Health Bio-Sciences (USA)). The copy number of the plasmid was calculated by the following equation: Copy number = (M × 6.022 × 10^23^)/(n × 650 × 1 × 10^9^), where M is the amount of DNA in nanograms, n is the length of the plasmid in base pairs, and the average weight of a base pair is 650 Daltons. The plasmid gradient dilution was from 10^8^ to 10^0^ copies/μl. RPA sensitivity was measured by the RPA reaction system, with each diluted concentration of positive plasmid as a template and RNase-free water as a negative control. The real-time PCR system measured the CRISPR/CAST package sensitivity by collecting fluorescent signals, and the nucleic acid test strip observed the color changes with the naked eye. Each concentration had three replicates.

### CRISPR/CAST package specificity test

Primers and crRNA sequences were compared with *Brucella* (GenBank No. CP025819, CP025820) and its cross-strains (*Yersinia enterocolitica* O:9*, Escherichia coli* O157*, Salmonella enterica serovar* Urbana O:30*,* and *Francisella tularensis*) by BLAST function. Apply the CRISPR/CAST package to assay simultaneously of *Brucella*, *Yersinia enterocolitica* O:9*, Escherichia coli* O157*, Salmonella enterica serovar* Urbana O:30*,* and *Francisella tularensis*, RNase-free water as a negative control for specificity test.

### Field assay of the CRISPR/CAST package for serum samples

The total number of serum samples was 498, including 398 samples of sheep serum and 100 samples of cattle serum from the Tongliao Zarut Banner area (April 2022), and *Brucella* DNA was extracted with a *Brucella* infection pretreatment kit (Zhai et al., 2017), wherein isolating and extracting the *Brucella* DNA comprises: taking 0.2 mL of serum into a 1.5 mL Ep tube; centrifuging at room temperature, 15000xg for 10 min to obtain a first pellet and a first supernatant; discarding the first supernatant; adding 0.2 mL of Solution V to the 1.5 mL Ep tube; shaking; centrifuging at room temperature, 15,000 × g for 10 min to obtain a second pellet and a second supernatant; discarding the second supernatant; adding 20 μL of Solution V to the 1.5 mL Ep tube; shaking, and instantaneously centrifuging; heating at 100 °C for 5 min; placing in ice or in a -20 °C refrigerator for 2 min; centrifuging at room temperature, 15000xg for 10 s to obtain a third supernatant. sucking 15 or 10 μL of the third supernatant as a template for the next assay, referring to the Bio-Lifesci Cas12/13 special nucleic acid test note instructions to interpret the results. The results were also compared with TaqMan probe-based real-time PCR [27] and RBT. The forward primer (bcsp31-F, ACCTTGCCCTTGCCATCAT), reverse primer (bcsp31-R, AGTCCGGCTTTACGCAGTCA) and probe (bcsp31-P, FAM-TGCCGTTATAGGCCCAATAGGCAACG- BHQ1) targeted *bcsp31* of *Brucella*. Primers and probes were used at a final concentration of 0.2 μM with 2 × Premix Ex Taq™ Probe qPCR (TaKaRa Bio Inc. Dalian, China) and 2 μl blood DNA in a 25 μl qPCR. The qPCR cycling conditions consisted of 95 °C for 30 s, followed by 45 cycles at 95 °C for 15 s and 60 °C for 30 s.

### Statistical analysis

SPSS 17.0 was employed to process the data. The data were analyzed using the homogeneity test of variance and normality test. Measurement data are expressed as the mean ± SEM. Univariates between groups were analyzed using the t test. Data among groups were analyzed by ANOVA. Heterogeneity of variance was analyzed using a nonparametric test. *P* < 0.05 was considered statistically significant.

### Supplementary Information

**Additional file 1: Figure S1.** crRNA screen. **Figure S2.** Sensitivity of the standard RPA for nucleic acid detection.

## Data Availability

The datasets generated and analysed during the current study are available in GenBank repository. GenBank No. OQ378327. https://www.ncbi.nlm.nih.gov/nuccore/OQ378327.

## Abbreviations

CRISPR: Clustered Regularly Interspaced Short Palindromic Repeats
Cas12a: CRISPR associated protein 12a
CRISPR/CAST: CRISPR/Cas12a Test
RPA: Recombinase polymerase amplification
RBT: Rose bengal test
PCR: Polymerase chain reaction
DETECTR: DNA endonuclease targeted CRISPR trans reporter
SHERLOCKv2: Specific High-sensitivity Enzymatic Reporter UnLOCKing Version 2
PAM: Protospacer-adjacent motif
6-AM: 6-carboxyfluorescein
FITC: Fluorescein isothiocyanate
LAMP: Loop-mediated isothermal amplification
RAA: Recombinase aided amplification
ssDNA: Single stranded DNA
crRNA: CRISPR-derived RNA
sgRNA: Small guide RNA
tracrRNA: Trans-activating RNA
ELISA: Enzyme-linked immunosorbent assay
