# Supplementary material for: CRISPR-Cas12a test strip (CRISPR/CAST) package: In-situ detection of Brucella from infected livestock
Source: BMC Vet Res. 2023 Oct 13;19:202. doi: 10.1186/s12917-023-03767-1 (PMC10571365; doi:10.1186/s12917-023-03767-1)
Supplement: Supplementary file 1 — Additional file 1: Figure S1. crRNA screen. Figure S2. Sensitivity of the standard RPA for nucleic acid detection. [file 12917_2023_3767_MOESM1_ESM.pdf]

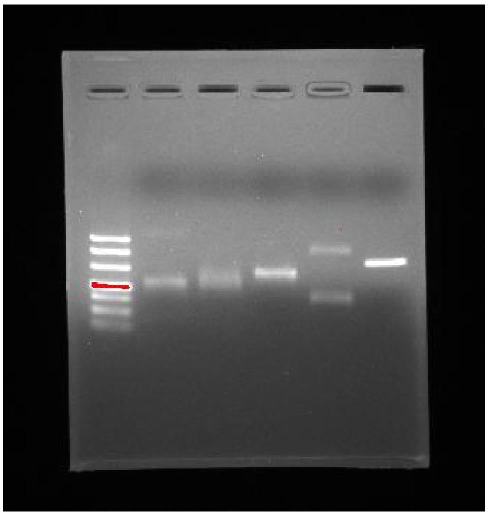

Figure S1 crRNA screen

Figure S1 showed crRNA screen on a 2% agarose gel electrophoresis. Lane 1: DL 500 DNA Marker (Takara, Code No.3590Q); Lane 2-5: RPA amplification products (340 bp) cleavage by Cas12a; Lane 2: 261/79 for crRNA-1; Lane 3: 303/37 for crRNA-2; Lane 4: 310/30 for crRNA-3; Lane 5: 174/166 for crRNA-4; Lane 6: Negative control. **Figure S1 is the original electrophorogram of Figure 3B in the manuscript.**

Instrument: Gel Doc^{TM} XR+ (Bio-RAD, U.S.A.)

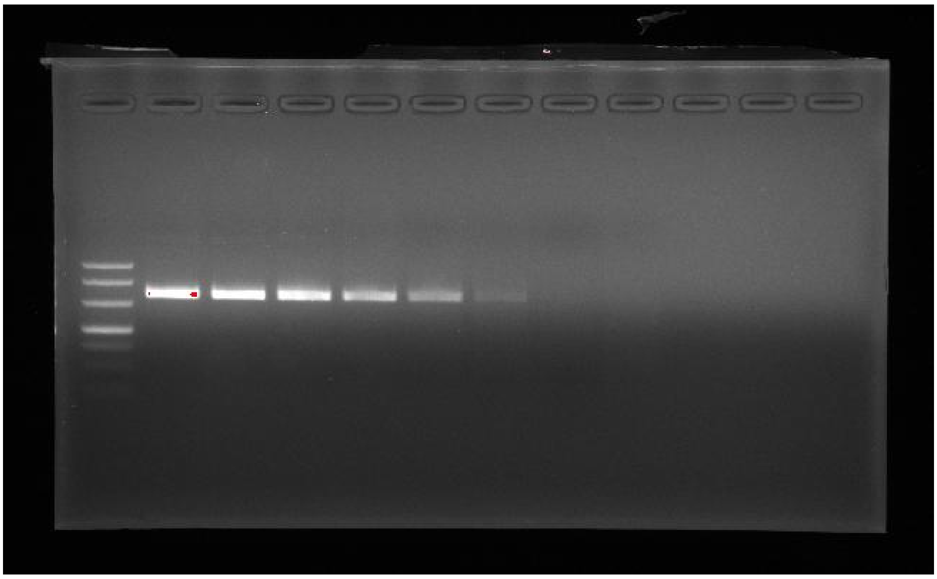

Figure S2 Sensitivity of the standard RPA for nucleic acid detection

Figure S2 showed the standard RPA sensitivity on 2% agarose gel electrophoresis. Lane 1: DL 500 DNA Marker (Takara, Code No.3590Q); Lane 2-10 corresponds to the copies of plasmid standard were 10^8-10^0 copies/μl; Lane 11: Negative control.

Figure S2 is the original electrophorogram of Figure 4A in the manuscript. We adopted " image-invert data" to acquire a grey background and black strip and removed the 12th lane because it is an unsampled lane.

Instrument: Gel Doc™ XR+ (Bio-RAD, U.S.A.)
